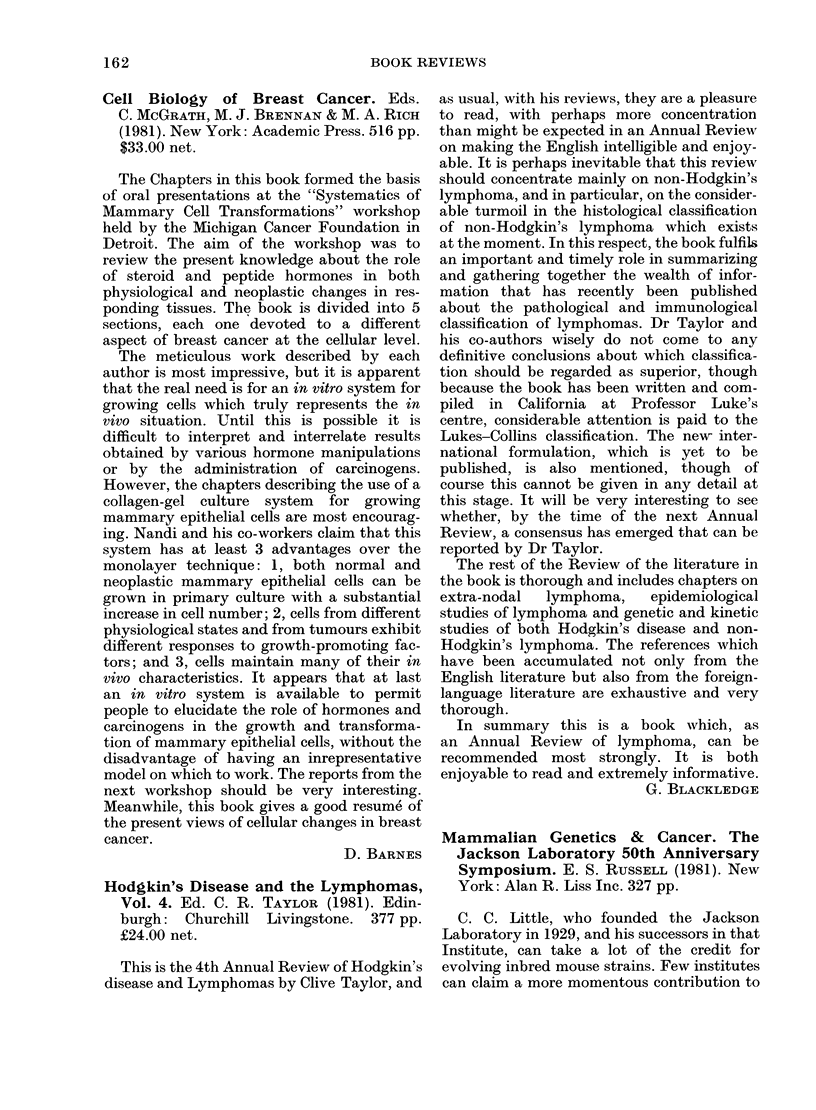# Cell Biology of Breast Cancer

**Published:** 1982-01

**Authors:** D. Barnes


**Cell Biology of Breast Cancer.** Eds. C. McGrath, M. J. Brennan & M. A. Rich (1981). New York: Academic Press. 516 pp. $33.00 net.

The Chapters in this book formed the basis of oral presentations at the "Systematics of Mammary Cell Transformations" workshop held by the Michigan Cancer Foundation in Detroit. The aim of the workshop was to review the present knowledge about the role of steroid and peptide hormones in both physiological and neoplastic changes in responding tissues. The book is divided into 5 sections, each one devoted to a different aspect of breast cancer at the cellular level.

The meticulous work described by each author is most impressive, but it is apparent that the real need is for an *in vitro* system for growing cells which truly represents the *in vivo* situation. Until this is possible it is difficult to interpret and interrelate results obtained by various hormone manipulations or by the administration of carcinogens. However, the chapters describing the use of a collagen-gel culture system for growing mammary epithelial cells are most encouraging. Nandi and his co-workers claim that this system has at least 3 advantages over the monolayer technique: 1, both normal and neoplastic mammary epithelial cells can be grown in primary culture with a substantial increase in cell number; 2, cells from different physiological states and from tumours exhibit different responses to growth-promoting factors; and 3, cells maintain many of their *in vivo* characteristics. It appears that at last an *in vitro* system is available to permit people to elucidate the role of hormones and carcinogens in the growth and transformation of mammary epithelial cells, without the disadvantage of having an inrepresentative model on which to work. The reports from the next workshop should be very interesting. Meanwhile, this book gives a good resumé of the present views of cellular changes in breast cancer.

D. Barnes